# The influence of body image on psychological symptomatology in breast cancer women undergoing intervention: a pre-post study

**DOI:** 10.3389/fpsyg.2024.1409538

**Published:** 2024-06-17

**Authors:** Veronica Verri, Ilaria Pepe, Chiara Abbatantuono, Morena Bottalico, Cristina Semeraro, Marco Moschetta, Maria Fara De Caro, Paolo Taurisano, Linda Antonella Antonucci, Alessandro Taurino

**Affiliations:** ^1^Department of Education, Psychology, Communication (For.Psi.Com.), University of Bari Aldo Moro, Bari, Italy; ^2^Department of Translational Biomedicine and Neuroscience (DiBraiN), University of Bari Aldo Moro, Bari, Italy; ^3^Interdisciplinary Department of Medicine (D.I.M.), Breast Care Unit, University of Bari Aldo Moro, Bari, Italy

**Keywords:** psycho-oncology, body image, breast cancer, pre−/post-surgery, psychological symptomatology

## Abstract

**Introduction:**

Body image concerns related to breast cancer surgery may challenge patients’ quality of life and their treatment outcomes, thus representing a key aspect to be assessed in the psycho-oncological settings. The present longitudinal study is aimed to (1) investigate the association between preoperative body image and postoperative psychological symptoms in breast cancer patients; (2) explore the impact of pre−/post-surgery variation in body image on psychological symptomatology.

**Methods:**

*N* = 72 women undergoing breast cancer surgery were preoperatively screened (T1) using the Body Uneasiness Test (BUT) and were assessed postoperatively (T2) using the Symptom Checklist-90 Revised (SCL-90-R) and re-administered the BUT. Spearman’s correlation was used to investigate the relationship between age, preoperative body image and postoperative psychological symptoms, and variation in body image. To predict post-surgical psychological symptomatology, two separated multiple regression models were used to evaluate preoperative body image and its variation after surgery controlling for covariates (i.e., education; intervention type). P significance was set as 0.05 for all analyses and adjusted for multiple comparisons.

**Results:**

At T1, anxiety in relation to body image scores emerged as the most frequently experienced psychological symptomatology after surgery (all adjusted *p* < 0.05). Significant correlations were observed between all SCL-90-R scores at T2 and avoidance behaviors and depersonalization scores at T1. The associations were most significantly strong for somatization, depression, anxiety, and hostility (all adjusted *p* < 0.05). However, change in body image between pre- and post-intervention was not associated with psychological symptomatology at T2 (all adjusted *p* > 0.05). Pre-surgery body avoidance was significantly associated with post-intervention psychological symptoms (SOMβ = 0.453, *p* = 0.0001; DEPβ = 0.507, *p* = 0.0001; AXβ = 0.459, *p* = 0.0001; HOSβ = 0.410, *p*=. 0001). However, increased weight phobia between pre- and post-surgery was statistically associated with increased somatization, anxiety, depression and hostility at T2 (βSOM = 0.439, *p* = 0.0001; βDEP = 0.454, *p* = 0.0001; βANX = 0.471, *p* = 0.0001).

**Discussion:**

Overall, pre−/post-intervention body concerns were significantly associated with primary psychological symptoms in breast cancer patients undergoing surgery. Higher levels of body avoidance and weight phobia were significantly associated with the primary psychological dimensions assessed. As body concerns might act as quality-of-life predictors, their evaluation is crucial in fostering patients’ well-being and treatment adherence.

## 1 Introduction

Breast cancer is the most common neoplasm worldwide (Zhang et al., 2021). It occurs predominantly in the female population, representing the leading cause of cancer death in women (Siegel et al., 2015). Early diagnosis and therapies used to treat the malignancy, such as surgery, chemotherapy, radiotherapy and hormone therapy, have contributed to increasing survival rates (Guedes et al., 2018). However, the side effects of these treatments can alter body image. For instance, surgery can cause appearance-related effects such as acute and chronic pain, anatomic changes, breast asymmetry, and loss of sensation, which can affect how women with breast cancer cope with their bodily changes (Lovelace et al., 2019), contributing to the development of negative body image (Boquiren et al., 2016).

Body image in the psychological context can also be related to the construct of the physical self (Cash and Pruzinsky, 2002). The concept of self can be defined more broadly as a three-dimensional image that each person has of him or herself (Salerno et al., 2017), or as the sum of several identity factors (including the body among the material ones), emotions, feelings, and the actions that drive self-seeking and self-preservation (James, 2011). Bodily changes may impact development and, in turn, bodily self-representations can positively influence decision-making processes by providing a protective sense of cohesion (Sebri et al., 2021). In this view, self-representations are involved in the way people perceive negative events, such as cancer diagnosis, by activating emotional and cognitive coping processes.

Body image can be defined as a multidimensional concept involving positive and negative perceptions, thoughts, feelings, and types of behavior about the whole body and its functioning (Vani et al., 2021). This construct is characterized by two key factors, i.e., the *body image evaluation* related to one’s satisfaction with his/her physical appearance, and *body image investment*, regarding the value assumed by physical attributes (Cash, 2012). Body image and its factors should be assessed in the oncological settings (Ong et al., 2017) as the relationship between body image and cancer is crucial to evaluate how patients experience both perceived and actual changes associated with the disease (White, 2000). This view emphasizes the importance of the individual’s perspective, regardless of whether the body changes are perceived by others.

Several studies investigated the relationship between breast cancer and body image, showing that women’s experiences during and after surgery can cause psychosocial changes (Davis et al., 2020). In particular, surgery is one event that might challenge the quality of life in breast cancer patients (Dell'Antônio Pereira et al., 2017). Traumatic experiences, including needing surgery, can consequently have an impact on one’s perception of one’s body, and this has been linked to a number of significant issues with body image (Chen et al., 2012). A poor body image was found to be positively correlated with the development of depressive symptoms, a lower quality of life, and the manifestation of interpersonal difficulties in breast cancer survivors (Begovic-Juhant et al., 2012). A recent study demonstrated that a negative body image could be associated with negative thoughts and feelings that may lead to engaging in maladaptive or health-risk types of behaviors in women diagnosed with breast cancer (Brunet et al., 2022). In addition, breast loss may dramatically impact the intimacy and self-perception of women with breast cancer, as shown in the study by Sun et al. (2018). Women who underwent surgery, particularly mastectomy, experienced a discrepancy between their self-image and that of society. Such discrepancy not only led to discomfort but also entailed feeling apart and an attitude of detachment from what was one’s bodily self-consciousness and relationships with others. For instance, for many women with breast cancer who had undergone mastectomy, the breast was seen as something disfigured that needed to be hidden from public view. These feelings have major repercussions on women’s daily lives, requiring special attention and acceptance from healthcare professionals (Sun et al., 2018).

Having a negative body image can also affect illness (Han et al., 2010). In a sample of patients aged from 31 to 45 years, body image dissatisfaction acted as a potential risk factor for physical and mental health (Phoosuwan and Lundberg, 2023). Additionally, undergoing surgery (e.g., quadrantectomy or mastectomy) can lead to the onset of anxiety-depressive symptoms (Davis et al., 2020). However, it should be noted that higher levels of satisfaction with physical appearance following treatment are associated with lower depression and shame levels (Peled et al., 2014). Body image dissatisfaction, especially in patients who have undergone invasive surgery, can also lead to somatosensory amplification, i.e., the tendency to experience somatic sensations as more intense or disturbing (Sertoz et al., 2009). Negative body image may increase the amount of dysmorphic, obsessive-compulsive types of behavior and thoughts (Sadri Damirch et al., 2019), causing embarrassment, mental conflict, hopelessness, and rumination (Kollei et al., 2012). In contrast, a positive self-perception of body could reduce psychological distress in women with breast cancer (Sherman et al., 2017).

In this regard, it is crucial to investigate the impact of body image on the different psychological dimensions of patients in order to target therapeutic options based on the possible risk or protective factors of women with breast cancer. Considering the importance of body image evaluation in psycho-oncology, data from a longitudinal cohort of women undergoing breast cancer surgery was used in this study to examine the relationship between different aspects of body image before the surgery and the mental health observed after the intervention procedure.

The present study aimed to multifariously investigate the relationship between body image and psychological outcomes in the context of breast cancer surgery. Specifically, the study pursued two specific goals: 1. Investigating the overall impact of baseline body image on the psychological consequences of breast surgery. 2. Investigating how variations in body image between the pre- and post-intervention time points affect the psychological effects of breast surgery. In particular, the perceived extent of the alteration in body image and its correlation with psychological symptoms following surgery were examined. The first hypothesis postulated that women with a more negative baseline body image would experience elevated levels of psychological distress, including anxiety, depression, somatization, and hostility after surgery. It was predicted that a more negative baseline body image would be associated with worse post-surgery psychological outcomes. By investigating this relationship, the study aimed to shed light on the potential impact of pre-existing perceptions of the bodily self on psychological well-being during the postoperative period.

The second hypothesis claimed that notable alterations in an individual’s perception of their body following surgery would be linked to commensurate modifications in their psychological well-being. It was also expected that women who had more significant changes in their perception of their bodies following surgery would be more psychologically distressed. The study sought to clarify the psychological effects of surgically altered body image and their influence on postoperative psychological status by examining this relationship.

## 2 Materials and methods

### 2.1 Participants

The study was conducted at the Breast Care Unit of Bari University Hospital in collaboration with the Senology Medical Team. The psychological assessment of breast cancer patients, consisting of a clinical interview and a psychological battery, was administered by qualified psychologists at two different time points, i.e., one week before surgery (T1) and two months after surgery (T2). Specifically, these time points coincided with pre-surgery admission to the hospital (T1) and a two-month follow-up for further medical examinations (T2). Exclusion criteria were: age under 18 years; presence of current or history of psychiatric disorders as described by the DSM-5 categorical system (APA, 2013); history of substance abuse; current alcohol or drug use; diagnosed medical comorbidities. The research protocol was previously approved by the Bari University Hospital’s local ethical committee (study code = ET-23-01) and complied with the 1964 Helsinki Declaration and later amendments. All participants provided written informed consent to enter the study.

### 2.2 Psychological assessment

During the clinical interview each patient was informed about the research purposes and procedures and provided written consent to enter the study. Following the Research Topic of socio-anamnestic and clinical information, psychological assessment was conducted first (T1) in the preoperative inpatient setting, and second (T2), in the postoperative setting, i.e., two months after surgery. The psychological variables, measured by appropriate psychometric tools, were preoperative and postoperative body image (T1 and T2) and postoperative primary symptomatic dimensions (T2).

To assess body image was used the *Body Uneasiness Test* (BUT; Cuzzolaro et al., 2006). It was used as it is considered a reliable and multidimensional tool to evaluate body image distress or concerns (Marano et al., 2007). It is a self-administered test divided into two parts. The first part (BUT-1) consists of 34 items. The total mean score of these items indicates the severity of one’s body image. The 34 items correspond to the 34 expressions commonly occurring in people with body dysmorphic disorder. Five areas were identified: (1) weight phobia (WP); (2) body image concerns (BIC); (3) avoidance behavior (A); (4) compulsive self-monitoring (CSM); and (5) depersonalization (D). The level of severity is expressed on a Likert scale from 0 to 5, where 0 means no problem and 5 means maximum severity. The second part of the test (BUT 2) consists of 37 items, each focused on a specific area of the body and sensory manifestations such as sweating, redness, odor, and noise. The items correspond to the 37 body parts or functions that are most problematic for people with body dysmorphic disorder. Overall, the higher the total score (BUT total), the greater the impairment in body image.

Additionally, we used the *Symptom Checklist Revised* (SCL-90-R; Derogatis, 1977; Preti et al., 2011) to assess primary symptomatic dimensions, which is a broad-spectrum instrument. The SCL-90-R is a self-report questionnaire consisting of 90 items referring to the psychological symptoms experienced last week. It was used to analyze nine primary symptomatic dimensions: (1) somatization; (2) obsessiveness; (3) interpersonal hypersensitivity; (4) depression; (5) anxiety; (6) hostility; (7) phobic anxiety; (8) paranoid ideation; (9) psychoticism. Three global indices complete the assessment: the *Global Severity Index* (GSI), which is a global indicator of the intensity of the psychological symptomatology reported by the subject; the *Positive Symptom Total* (PST), which concerns the number of symptoms reported by the subject; and the *Positive Symptom Distress Index* (PSDI), which is used as an index of response style. The SCL-90-R has been shown to be a reliable multidimensional scale (Paap et al., 2011), and the nine dimensions have been extensively validated in a large patient population (Derogatis et al., 1970; Dinning and Evans, 1977; Clark and Friedman, 1983).

Measuring psychological indices using the SCL-90-R, only those most frequently linked to body image in relation to breast cancer and surgical procedures were selected for analysis (Bastecký et al., 1996; Pan et al., 2014; Carreira et al., 2018). Specifically, the dimensions most frequently examined in the analyzes included depression (Hsiao et al., 2019), somatization (Leonhart et al., 2017), anxiety (Hashemi et al., 2020; Botto et al., 2022) and hostility.(Martino et al., 2019; Grassi et al., 2021). Thus, the inclusion of SCL-90 scores in the analysis design (see section 3.3) was limited to these four subscales.

Overall, both BUT and SCL-90-R show good psychometric properties as the levels of Cronbach’s alpha of the BUT range between 0.69 (only BUT-2, hair and skin items) and 0.90 (Cuzzolaro et al., 2006), while those of SCL-90-R range between 0.70 and 0.96 (Prunas et al., 2012).

### 2.3 Statistical analysis

Statistical analyses were performed with the SPSS 26.0 software. Descriptive statistics for demographic and clinical data were calculated using the mean and standard deviation for quantitative variables and the frequency and percentage for qualitative variables. To identify potential confounding variables, the effects of clinical and demographic parameters were evaluated. To determine whether age, marital status, level of education, and type of surgery had a significant impact on psychological outcomes, preliminary analyses were carried out. Differences in mean of SCL-90-R, T2 and BUT at T1, and BUT calculated as the difference between T2 and T1 for each potential confounding variable were examined using the Mann–Whitney U-test for two independent samples, the Kruskal-Wallis test for more than two independent samples, and the Wilcoxon test for dependent samples after determining the absence of normality according to the Kolmogorov–Smirnov test at *p*≤. 05.

To determine which variables were most strongly associated with the psychological side effects experienced following surgery, a two-stage analysis was conducted.

#### 2.3.1 Psychological correlates of body image

To find out if there was a relationship between the correlates of baseline body image, age (a potential covariate) and psychological symptomatology, as well as between the change in body image after the surgical procedure, age and symptomatology, a first step involved using the Spearman correlation adjusted with the Benjamini-Hochberg correction for multiple testing.

#### 2.3.2 Body image as a predictor of post-surgery psychic symptoms

In the second step of analysis, separate multiple linear regression models were constructed to identify significant determinants of psychological symptomatology. Initially, perceived body image before surgery was considered the independent variable, with somatization, anxiety, depression, and hostility experienced after surgery as dependent variables. Subsequently, the change in body image between pre- and post-operative periods was considered the independent variable, while psychological symptoms were treated as the dependent variable. As dummy covariates of interest, level of education (high school or higher = 0, secondary school/other = 1) in the first models and type of surgical intervention (0 = mastectomy, 1 = quadrantectomy) in the second models were included. Variance inflation factor (VIF) was used to assess multicollinearity. After adjusting for the Benjamini-Hochberg correction, a two-tailed *p*-value <0.05 indicated statistical significance for each model. For each outcome variable coefficient of determination (*R*^2^), and standardized regression coefficient (β) were provided to demonstrate the strength and degree of fit of the connection.

## 3 Results

### 3.1 Sample characteristics

Seventy-two patients were enrolled, with an average age of 51 (SD = 9.66; range 24–73). Table 1 indicates the mean, SD, and percentages for socio-demographic, clinical, and psychological variables.

**Table 1:** Demographic and clinical characteristics of the sample.

Variables	All patients (*n* = 72)	Mastectomy group (*n* = 26)	Quadrantectomy group (*n* = 46)	*p* value
Age in years, *mean* (SD)	51.04 (9.66)	48.88 (10.49)	52.26 (9.05)	0.087^a^
Marital status *n* (%)				0.93^b^
Single	10 (25.1)	2 (7.7)	8 (17.4)	
Divorced/widowed	9 (31.8)	5 (23.1)	4 (8.7)	
Married	53 (73.6)	19 (73.1)	34 (73.9)	
Education *n* (%)				0.31^b^
Higher scholar grade	27 (37.5)	12 (46.2)	15 (32.6)	
Low/secondary scholar grade	45 (62.5)	14 (53.8)	31 (67.4)	
Familiarity with the disease *n* (%)				0.75^b^
Yes	37 (51.4)	12 (46.2)	23 (50.0)	
No	35 (48.6)	14 (53.8)	23(50.0)	

^a^Mann Withney Test.^b^Chi-square test.*p* value < 0.05 in bold was statistically significant.

The mean age of mastectomy patients did not differ from quadrantectomy patients. (48.88 ± 10.49 vs. 52.26 ± 9.05; U = 744.0; *p* = 0.087) nor did years of education (*χ*^2^ = 0.314; *p* = 0.31), marital status between the two groups (*χ*^2^ = 0.365; *p* = 0.93).

While there were no differences in BUT scores at T1 based on marital status, type of surgery, or level of education, there were statistically significant differences between education levels regarding of total BUT scores (*U* = 488, *p* = 0.03). There was no significant difference in pre-post-intervention BUT scores or scores at T2 in relation to the four SCL-90-R indices in any of the clinical/sociodemographic variables. There were no statistically significant differences in body image scores before and after the surgery. Tables 2–4 shows the test statistics and corresponding *p*-values.

**Table 2:** Comparative analysis of body image and psychological symptoms at different time points by type of surgery.

Variables	Type of surgery						
	*U*	*p*	*r*	Median		Interquartile range	
				Mastectomy	Quadrantectomy	Mastectomy	Quadrantectomy
BUT baseline							
WP	564	0.68	0.7	1.06	1.06	1.88	1.09
BIC	573	0.77	0.7	0.94	1.22	1.33	1.22
A	461	0.10	0.1	0.91	0.67	1.13	1.00
CSM	551	0.57	0.6	1.17	1.00	1.17	1.46
D	536	0.45	0.5	0.90	0.40	1.15	1.00
Total score	566	0.71	0.7	41.00	34.00	36.50	29.30
ΔBUT (T2-T1)							
ΔWP	546	0.54	0.09	0.00	0.00	1.31	0.81
ΔBIC	482	0.17	0.2	0.17	−0.11	1.33	0.56
ΔA	592	0.90	0.01	−0.17	0.00	1.25	0.67
ΔCSM	543	0.49	0.09	0.00	0.00	0.80	0.95
ΔD	580	0.87	0.03	0.00	0.00	1.00	0.60
ΔTotal score	540	0.49	0.09	0.00	−0.50	32.00	25.80
SCL-90 R T2							
SOM	477	0.15	0.2	59.3	57.3	23.6	23.0
DEP	481	0.17	0.2	54.4	52.1	34.5	18.1
ANX	502	0.26	0.2	52.3	49.0	35.0	15.8
HOS	518	0.34	0.1	45.5	49.7	24.3	13.9

*U*, Mann Withney test statistics; *p*, *p*-value; *r*, effect size (rank biserial correlation); *p*-value < 0.05 in bold was statistically significant.

BUT, body uneasiness test; WF, weight phobia; BIC, body image concern; A, avoidance behavior; CSM, compulsive self-monitoring; D, depersonalization.

ΔBUT (T2-T1), boby uneasiness test variation (T2 = post-surgery- T1 = pre-surgery); ΔWP, weight phobia variation; ΔBIC, body image concern variation; ΔA, avoidance behavior variation; ΔCSM, compulsive self-monitoring variation; ΔD, depersonalization variation; ΔTotal score, total score variation.

SCL-90 R T2, symptom checklist revised post-surgery; SOM, somatization; DEP, depression; ANX, anxiety; HOS, hostility.

**Table 3:** Comparative analysis of body image and psychological symptoms at different time points by educational level.

Variables	Education						
	*U*	*p*	*r*	Median		Interquartile	Range
				Low/secondary scholar grade	Higher scholar grade	Low/secondary scholar grade	Higher scholar grade
BUT baseline							
WP	525	0.06	0.10	1.00	1.13	1.69	1.00
BIC	514	0.14	0.2	1.00	1.22	1.06	1.22
A	559	0.12	0.08	0.50	0.83	0.91	1.00
CSM	460	0.07	0.2	0.50	1.17	1.25	1.00
D	489	0.21	0.2	0.20	0.60	1.00	1.00
Total score	488	**0.03**	0.2	28.00	36.00	35.50	30.00
ΔBUT (T2-T1)							
ΔWP	566	0.70	0.07	0.00	0.00	0.43	1.00
ΔBIC	562	0.70	0.08	0.00	−0.10	0.56	1.00
ΔA	548	0.63	0.1	0.00	0.00	1.00	0.83
ΔCSM	473	0.22	0.2	0.17	−0.17	0.67	1.00
ΔD	583	0.72	0.04	0.00	0.00	0.70	0.60
ΔTotal score	569	0.38	0.06	0.00	−1.00	26.50	27.00
SCL-90 R T2							
SOM	537	0.33	0.1	56.0	56.0	19.7	24.1
DEP	578	0.22	0.05	52.1	52.1	15.8	20.4
ANX	594	0.17	0.02	50.7	47.3	18.3	20.0
HOS	572	0.45	0.06	50.9	52.9	11.6	18.5

U, Mann Withney test statistics; *p*, *p*-value; *r*, effect size (rank biserial correlation); *p*-value < 0.05 in bold was statistically significant.

BUT, body uneasiness test; WF, weight phobia; BIC, body image concern; A, avoidance behavior. CSM, compulsive self-monitoring; D, depersonalization.

ΔBUT (T2-T1), boby uneasiness test variation (T2 = post-surgery- T1 = pre-surgery); ΔWP, weight phobia variation; ΔBIC, body image concern variation; ΔA, avoidance behavior variation; ΔCSM, compulsive self-monitoring variation; ΔD, depersonalization variation; ΔTotal score, total score variation.

SCL-90 R T2, symptom checklist revised post-surgery; SOM, somatization; DEP, depression; ANX, anxiety; HOS, hostility.

**Table 4:** Comparative analysis of body image and psychological symptoms at different time points by marital status.

Variables	Marital status								
	*H*	*p*	*η* ^2^	Median			Interquartile range		
				Single	Divorced/widowed	Married	Single	Divorced/widowed	Married
BUT baseline									
WP	5.56	0.35	0.08	1.25	1.13	1.00	1.06	1.50	1.38
BIC	6.35	0.27	0.09	1.17	1.56	1.11	0.94	1.44	1.22
A	4.88	0.43	0.07	0.41	1.00	0.67	1.13	1.17	1.00
CSM	7.73	0.17	0.10	1.17	1.17	0.83	0.75	1.33	1.50
D	4.88	0.43	0.07	0.70	1.00	0.20	1.10	1.20	1.00
Total score	5.92	0.31	0.08	42.50	43.00	33.00	11.80	52.00	34.00
ΔBUT (T2-T1)									
ΔWP	5.71	0.33	0.08	−0.19	0.25	−0.13	1.66	1.00	0.87
ΔBIC	3.07	0.69	0.04	−0.11	0.00	−0.11	1.19	0.55	0.66
ΔA	4.17	0.52	0.06	0.00	0.00	−0.17	0.50	1.00	0.83
ΔCSM	8.81	0.13	0.12	−0.08	0.50	0.00	0.87	0.83	0.83
ΔD	5.13	0.42	0.07	−0.30	0.00	0.00	0.75	0.40	0.60
ΔTotal score	5.74	0.67	0.08	0.00	10.00	−1.00	30.50	20.00	26.00
SCL-90 R T2									
SOM	8.87	0.11	0.13	61.4	62.5	49.4	18.6	39.5	21.9
DEP	3.65	0.60	0.05	48.7	58.9	52.1	17.5	43.0	18.1
ANX	5.36	0.37	0.08	54.0	60.7	47.3	21.7	43.3	16.7
HOS	6.30	0.28	0.09	45.5	45.5	45.5	11.6	27.8	18.5

H, Kruskal-Wallis Test Statistics; *p*, *p*-value; *η*^2^, effect size (eta-squared); *p*-value < 0.05 in bold was statistically significant.

BUT, body uneasiness test; WF, weight phobia; BIC, body image concern; A, avoidance behavior; CSM, compulsive self-monitoring; D, depersonalization.

ΔBUT (T2-T1), boby uneasiness test variation (T2 = post-surgery- T1 = pre-surgery); ΔWP, weight phobia variation; ΔBIC, body image concern variation; ΔA, avoidance behavior variation; ΔCSM, compulsive self-monitoring variation; ΔD, depersonalization variation; ΔTotal score, total score variation.

SCL-90 R T2, symptom checklist revised post-surgery; SOM, somatization; DEP, depression; ANX, anxiety; HOS, hostility.

### 3.2 Psychological correlates of baseline body image

Table 5 shows the bivariate correlations between the Body Uneasiness Test (BUT) scales assessed at T1, including the BUT summary and component scales, and the four indices derived from the Symptom Checklist-90 (SCL-90-R) administered after surgery, as well as age.

**Table 5:** Spearman’s correlations adjusted for multiple comparisons with the Benjamini-Hochberg procedure between baseline BUT score and post-surgery psychological responses in total sample.

	AGE	SCL-90-R SOM	SCL-90-R DEP	SCL-90 R ANX	SCL-90 R HOS
BUT WP					
Spearman correlation	−0.16	0.271	0.311	0.32	0.18
Sig. (2-tailed) with Benjamini-Hocberg correction	0.24	0.046*	0.021*	0.02*	0.18
N	72	72	72	72	72
BUT BIC					
Spearman correlation	−0.023	0.174	0.223	0.296	0.153
Sig. (2-tailed) with Benjamini-Hocberg correction	0.84	0.20	0.11	0.03*	0.24
N	72	72	72	72	72
BUT A					
Spearman correlation	−0.117	0.322	0.404	0.367	0.343
Sig. (2-tailed) with Benjamini-Hocberg correction	0.37	0.02*	0.000**	0.01**	0.02*
N	72	72	72	72	72
BUT CSM					
Spearman correlation	−0.241	0.314	0.326	0.374	0.206
Sig. (2-tailed) with Benjamini-Hocberg correction	0.08	0.02*	0.02*	0.01**	0.13
N	72	72	72	72	72
BUT D					
Spearman correlation	−0.079	0.346	0.363	0.378	0.328
Sig. (2-tailed) with Benjamini-Hocberg correction	0.53	0.02*	0.02*	0.01**	0.02*
N	72	72	72	72	72
BUT total score					
Spearman correlation	−0.127	0.278	0.318	0.341	0.217
Sig. (2-tailed) with Benjamini-Hocberg correction	0.33	0.04*	0.02*	0.02*	0.12
N	72	72	72	72	72

*p < 0.05. **p < 0.01 (two-tailed).

BUT, body uneasiness test; WP, weight phobia; BIC, body image concern; A, avoidance behavior; CSM, compulsive self-monitoring; D, depersonalization.

SCL-90 R T2, symptom checklist revised post-surgery; SOM, somatization; DEP, depression; ANX, anxiety; HOS, hostility.

Notably, age had no significant relationship with any of the variables examined (WP, *ρ* = −0.15; *p* = 0.24; BIC, *ρ* = −0.20; *p* = 0.85; A, *ρ* = −0.11 *p* = 0.37; CSM, *ρ* = −0.24 *p* = 0.37; D, *ρ* = −0.08; *p* = 0.53; Total, *ρ* = −0.13; *p* = 0.33). The study found strong associations between baseline body image disturbance scores and post-surgery psychological symptoms. Specifically, higher scores on the body avoidance (SOM, *ρ* = 0.32, *p* = 0.02; DEP, *ρ* = 0.40, *p* = 0.0001; ANX, *ρ* = 0.37, *p* = 0.015; HOS, *ρ* = 0.34, *p* = 0.02) and depersonalization scales (SOM, *ρ* = 0.35, *p* = 0.02;DEP, *ρ* = 0.36, *p* = 0.02; ANX, *ρ* = 0.38, *p* = 0.01; HOS, *ρ* = 0.33, *p* = 0.02) were significantly correlated with higher scores on all four SCL-90-R subscales.

Furthermore, the main factor that showed a positive correlation with increased pre-surgery body dysmorphism in each subcomponent was the post-surgery anxious symptoms (WP, *ρ* = 0.32; *p* = 0.02; BIC, *ρ* = 0.30; *p* = 0.03; A, *ρ* = 0.37, *p* = 0.01; CSM, *ρ* = 0.37 *p* = 0.01; D, *ρ* = 0.38; *p* = 0.01; Total, *ρ* = −0.34; *p* = 0.01).

Additionally, the overall impairment in body image, as measured by the total BUT score was associated with higher scores in Anxiety (*ρ* = 0.34, *p* = 0.01), Somatization (*ρ* = 0.28, *p* = 0.04), and post-surgery Depression (*ρ* = 0.33, *p* = 0.02). No such association was found with Hostility (*ρ* = 0.22, *p* = 0.12).

### 3.3 Psychological correlates of variation in body image between pre- and post-surgery

The correlation analyses conducted to explore the relationship between changes in body image from T1 to T2 and the subsequent psychological symptoms experienced by the participants did not yield any significant findings. Specifically, there was no observed association between the degree of change in body image resulting from the surgery and the subsequent increase in Anxiety, Somatization, Depression, or post-surgery Hostility (*p* > 0.05). Detailed statistical values can be found in Table 6.

**Table 6:** Spearman’s correlation adjusted for multiple comparisons with the Benjamini-Hochberg procedure between changes in BUT score between post and pre-surgery and post-surgery psychological responses in the total sample.

	AGE	SCL-90-R SOM	SCL-90-R DEP	SCL-90 R ANX	SCL-90 R HOS
BUT ΔWF					
Spearman correlation	0.002	0.268	0.205	0.256	0.213
Sig. (2-tailed) with Benjamini-Hocberg correction	0.98	0.29	0.33	0.29	0.33
N	72	72	72	72	72
BUT ΔBIC					
Spearman correlation	−0.009	0.23	0.231	0.21	0.145
Sig. (2-tailed) with Benjamini-Hocberg correction	0.96	0.29	0.29	0.33	0.52
N	72	72	72	72	72
BUT ΔA					
Spearman correlation	0.095	0.123	0.12	0.165	0.098
Sig. (2-tailed) with Benjamini-Hocberg correction	0.69	0.63	0.63	0.52	0.69
N	72	72	72	72	72
BUT ΔCSM					
Spearman correlation	0.01	0.158	0.098	0.122	0.148
Sig. (2-tailed) with Benjamini-Hocberg correction	0.69	0.52	0.69	0.63	0.52
N	72	72	72	72	72
BUT ΔD					
Spearman correlation	0.05	0.026	0.053	0.03	−0.029
Sig. (2-tailed) with Benjamini-Hocberg correction	0.90	0.92	0.90	0.92	0.92
N	72	72	72	72	72
BUT ΔTotal score					
Spearman correlation	0.057	0.264	0.241	0.247	0.201
Sig. (2-tailed) with Benjamini-Hocberg correction	0.90	0.30	0.30	0.30	0.33
N	72	72	72	72	72

*p < 0.05. **p < 0.01 (two-tailed).

ΔBUT (T2-T1), boby uneasiness test variation (T2 = post-surgery- T1 = pre-surgery); ΔWP, weight phobia variation; ΔBIC, body image concern variation; ΔA, avoidance behavior variation; ΔCSM, compulsive self-monitoring variation; ΔD, depersonalization variation; ΔTotal score, total score variation.

SCL-90 R T2, symptom checklist revised post-surgery; SOM, somatization; DEP, depression; ANX, anxiety; HOS, hostility.

### 3.4 Basic body image as a predictor of post-operative psychic symptoms

In each regression model predicting post-surgical psychological symptoms according to the five indices that reveal a disturbance in body image at baseline (WP, BIC, A, CSM, and D), and level of education as a covariate, approximately 20% of the variance was explained, and the results were statistically significant (*F*_1_ = 18.12, *p* = 0.0006; *F*_2_ = 24,18, *p* = 0.00002; *F*_3_ = 18.71, *p* = 0.0004; *F*_4_ = 14,12, *p* = 0.00009) (Table 7). Notably, avoidance behavior at T1 consistently showed a positive relationship with increased levels of Somatization (*β* = 0.453; *p* = 0.0001), Depression (*β* = 0.507; *p* = 0.0001), Anxiety (*β* = 0.459; *p* = 0.0001) and Hostility (*β* = 0.410; *p* = 0.0001) following surgery. Assessment of multicollinearity using Variance Inflation Factors (VIF) ranging from 1.08 to 6.22 revealed no significant issues in the model.

**Table 7:** Results of backward multiple linear regression adjusted for multiple comparisons with the Benjamini-Hochberg procedure between body image at baseline and selected SCL-90 R subscales at T2 and education as covariate (*n* = 72).

**Outcome**	**Predictors**	***B***	**SE**	***β***	***t*-value**	***p***	**Tolerance**	**VIF**
**Initial model 1**, *R*^2^ = 0.237, *F* = 3.37, Sig. = 0.006								
SOM	Education^a^	−2.004	4.526	−0.050	−0.443	0.659	0.931	1.075
	WF	1.803	3.874	0.094	0.466	0.643	0.290	3.443
	BIC	−6.792	5.140	−0.300	−1.321	0.191	0.228	4.388
	A	12.952	6.764	0.517	1.915	0.060	0.161	6.218
	CSM	3.118	4.026	0.136	0.775	0.441	0.382	2.620
	D	0.346	5.670	0.016	0.061	0.952	0.181	5.540
**Final model 1**, *R*^2^ = 0.257, *F* = 18.2, Sig. with Benjamini-Hocberg correction = 0.0006								
SOM	A	11.355	2.667	0.453	4.257	0.000		
**Initial model 2**, *R*^2^ = 0.282. *F* = 5.26. Sig. = 0.001								
DEP	Education^a^	1.387	4.815	0.031	0.288	0.774	0.931	1.075
	WF	−0.210	4.121	−0.010	−0.051	0.959	0.290	3.443
	BIC	−6.676	5.468	−0.269	−1.221	0.227	0.228	4.388
	A	15.449	7.196	0.563	2.147	0.036	0.161	6.218
	CSM	1.144	4.284	0.045	0.267	0.790	0.382	2.620
	D	3.439	6.032	0.141	0.570	0.571	0.181	5.540
**Final model 2**, *R*^2^ = 0.257, *F* = 24.18, Sig. with Benjamini-Hocberg correction = 0.000002								
DEP	A	13.915	2.830	0.507	4.918	0.000		
**Initial model 3**, *R*^2^ = 0.223, *F* = 3.11, Sig. = 0.010								
ANX	Education^a^	−1.139	5.711	−0.023	−0.199	0.842	0.931	1.075
	WF	−1.868	4.888	−0.078	−0.382	0.704	0.290	3.443
	BIC	−3.274	6.486	−0.116	−0.505	0.615	0.228	4.388
	A	15.560	8.535	0.497	1.823	0.073	0.161	6.218
	CSM	3.328	5.081	0.116	0.655	0.515	0.382	2.620
	D	0.807	7.154	0.029	0.113	0.911	0.181	5.540
**Final model 3** *R*^2^ = 0.211, *F* = 18.71, Sig. with Benjamini-Hocberg correction = 0.0004								
ANX	A	14.377	3.324	0.459	4.326	0.000		
**Initial model 4,** *R*^2^ = 0.199, *F* = 2.69, Sig. = 0.021								
HOS	Education^a^	1.231	3.509	0.040	0.351	0.727	0.931	1.075
	WF	−1.051	3.003	−0.072	−0.350	0.727	0.290	3.443
	BIC	−3.580	3.985	−0.209	−0.898	0.372	0.228	4.388
	A	9.865	5.243	0.521	1.881	0.064	0.161	6.218
	CSM	−0.706	3.121	−0.041	−0.226	0.822	0.382	2.620
	D	2.398	4.395	0.143	0.546	0.587	0.181	5.540
**Final model 4**, *R*^2^ = 0.168, *F* = 14.12, Sig. with Benjamini-Hocberg correction = 0.000009								
HOS	A	7.760	2.065	0.410	3.757	0.000		

B, unstandardized coefficients; SE, standard error; Beta (β), standardized coefficients; *p*, *p*-value; VIF, variance inflation factor; *R*^2^, R-squared; *F*, *F*-Test; Sig., significance; Education^a^, (high school or higher = 0, secondary school/other = 1); WP, weight phobia; BIC, body image concern; A, avoidance behavior. CSM, compulsive self-monitoring; D, depersonalization; SOM, somatization; DEP, depression; ANX, anxiety; HOS, hostility.

### 3.5 Perceived change in body image after surgery as a predictor of postoperative psychological symptoms

The regression models that investigated the impact of perceived body image changes from pre- to post-surgery on psychological symptoms in women undergoing breast surgery net of the type of surgery, produced significant results (refer to Table 8). Each analysis was statistically significant, with *F*_1_ = 16.691, *p* = 0.0016; *F*_2_ = 18.18, *p* = 0.001; *F*_3_ = 19.92, *p* = 0.001; *F*_4_ = 17,14, *p* = 0.001. All five independent variables (ΔWP, ΔBIC, ΔA, ΔCSM, ΔD) 2 explained roughly 20% of the variance in symptoms, with no evidence of collinearity (Variance Inflation Factors <10). The observed dependence on increased weight phobia and heightened psychological symptoms post-surgery was specifically significant [(βSOM) =0.439; *p* = 0.0001; β (DEP) = 0.454; *p* = 0.0001; β (ANX) = 0.471; *p* = 0.0001; β (HOS) = 0.446; *p* = 0.0001].

**Table 8:** Results of backward multiple linear regression adjusted for multiple comparisons with the Benjamini-Hochberg procedure between change in body image between post and pre-surgery and selected SCL-90 R subscales at T2 and type of surgery as covariate (*n* = 72).

**Outcome**	**Predictors**	***B***	**SE**	***β***	***t*-value**	***p***	**Tolerance**	**VIF**
**Initial model 1,** *R*^2^ = 0.225. *F* = 3.14. Sig. = 0.009								
SOM	Type of surgery^b^	−4.296	4.633	−0.106	−0.927	0.357	0.917	1.091
	ΔWP	5.449	4.065	0.244	1.340	0.185	0.360	2.779
	ΔBIC	3.484	4.320	0.158	0.806	0.423	0.309	3.232
	ΔA	2.866	4.590	0.120	0.624	0.535	0.323	3.092
	ΔCSM	−0.065	3.478	−0.003	−0.019	0.985	0.580	1.723
	ΔD	−1.090	4.417	−0.045	−0.247	0.806	0.354	2.826
**Final Model 1,** *R*^2^ = 0.193. *F* = 16.69. Sig. with Benjamini-Hocberg correction = 0.0016								
SOM	ΔWP	9.800	2.399	0.439	4.085	0.000		
**Initial model 2,** *R*^2^ = 0.259. *F* = 3.78. Sig. = 0.003								
DEP	Type of surgery^b^	−3.330	4.970	−0.075	−0.670	0.505	0.917	1.091
	ΔWP	5.767	4.361	0.235	1.323	0.191	0.360	2.779
	ΔBIC	5.960	4.634	0.247	1.286	0.203	0.309	3.232
	ΔA	6.069	4.924	0.231	1.233	0.222	0.323	3.092
	ΔCSM	−1.171	3.730	−0.044	−0.314	0.755	0.580	1.723
	ΔD	−5.526	4.737	−0.209	−1.166	0.248	0.354	2.826
**Final model 2**, *R*^2^ = 0.206. *F* = 18.18. Sig. with Benjamini-Hocberg correction = 0.0011								
DEP	ΔWP	11.122	2.608	0.454	4.264	0.000		
**Initial model 3**, *R*^2^ = 0.258. *F* = 3.77. Sig. = 0.003								
ANX	Type of surgery^b^	−4.422	5.667	−0.087	−0.780	0.438	0.917	1.091
	ΔWP	7.276	4.972	0.261	1.463	0.148	0.360	2.779
	ΔBIC	4.115	5.284	0.150	0.779	0.439	0.309	3.232
	ΔA	5.248	5.615	0.176	0.935	0.353	0.323	3.092
	ΔCSM	1.456	4.254	0.048	0.342	0.733	0.580	1.723
	ΔD	−3.268	5.402	−0.109	−0.605	0.547	0.354	2.826
**Final model 3,** *R*^2^ = 0.222. *F* = 19.92. Sig. with Benjamini-Hocberg correction = 0.0011								
ANX	ΔWP	13.142	2.945	0.471	4.463	0.000		
**Initial model 4,** *R*^2^ = 0.192. *F* = 3.81. Sig. = 0.003								
HOS	Type of surgery^b^	−3.915	3.423	−0.127	−1.144	0.257	0.917	1.091
	ΔWP	3.019	3.004	0.179	1.005	0.319	0.360	2.779
	ΔBIC	2.555	3.192	0.153	0.800	0.426	0.309	3.232
	ΔA	4.179	3.392	0.231	1.232	0.222	0.323	3.092
	ΔCSM	1.113	2.570	0.061	0.433	0.666	0.580	1.723
	ΔD	−1.922	3.263	−0.106	−0.589	0.558	0.354	2.826
**Final model 4**, *R*^2^ = 0.199. *F* = 17.41. Sig. with Benjamini-Hocberg correction = 0.0011								
HOS	ΔWP	7.540	1.807	0.446	4.173	0.000		

B, unstandardized coefficients; SE, standard error; Beta (β), standardized coefficients; *p*, *p*-value; VIF, variance inflation factor; *R*^2^, R-squared; *F*, *F*-Test; Sig., Significance; Type of surgery^b^, (0 = mastectomy, 1 = quadrantectomy); ΔWP, weight phobia variation; ΔBIC, body image concern variation; ΔA, avoidance behavior variation; ΔCSM, compulsive self-monitoring variation; ΔD, depersonalization variation; SOM, somatization; DEP, depression; ANX, anxiety; HOS, hostility.

## 4 Discussion

The results of our study showed the influence of body image on primary psychological symptomatology in women with breast cancer who underwent surgery. The psychological journey of these patients encompasses various facets, from preoperative body concerns to postoperative psychological distress. Our study examined this complex relationship, highlighting the role that body image plays in the emergence of psychological outcomes both before and after surgery. Body image alteration is indeed a very relevant psychosocial problem for women with breast cancer (Pierrisnard et al., 2018). Body image is continuously shaped by perceptual, emotional, and cognitive-behavioral processes, and includes a ‘real body’, a ‘perceived body’, an ‘ideal body’ and a ‘socially accepted body’ (Schilder, 1935; Fiori and Giannetti, 2009). The experience of body image in women with breast cancer is a dynamic phenomenon involving numerous changes and alterations (Rodrigues et al., 2023).

The first aim of our study was to investigate baseline body image in the pre-surgical period: body avoidance in the pre-surgical period was positively associated with symptoms related to anxiety, depression, somatization and hostility. Avoiding one’s body might make it harder to interact with others and make one feel less satisfied with their body (Welsch et al., 2020). A negative assessment of one’s body, or a difference between one’s ideal body and perceived body, is known as body dissatisfaction (Stice and Shaw, 2002; Neves et al., 2017) and it has been linked to increased levels of psychological distress (Griffiths et al., 2016). ‘Body avoidance’ specifically refers to efforts to avoid, suppress, modify or escape stressful and negative thoughts, feelings, and sensations about the body (Timko et al., 2014). Such efforts are reflected by behaviors such as covering mirrors, wearing oversized clothing, and refusing to be weighed (Fairburn et al., 2003). It has been observed that lower body esteem scores, which can lead to avoidant behavior, are linked to higher levels of depression, anxiety, and somatization (Biby, 1998; Cohen et al., 2011). The term somatization refers to a condition of psychological distress in which personal distress is expressed through physical symptoms in the absence of any underlying organic cause (Kleinman and Good, 1985). It has been found that, in the context of individuals suffering from different oncological types, the most prominent somatic symptoms are tremor, lethargy, dizziness, tiredness, poor concentration and irritability (Cella and Tross, 1986; Maguire, 1999; Grassi et al., 2013). Other common somatic complaints related to cancer were pain, fatigue, and sensory symptoms (Chaturvedi et al., 1993). It was hypnotized by Carlson et al. (2004) that somatization was more frequent in cancer patients due to the high prevalence of psychological distress. Breast cancer may have a negative effect on body image, leading to body avoidance, sexual function, and guilt that may result in somatization as a cry for help, to express treatment fatigue (Chaturvedi et al., 2006). In a study conducted by De Luca et al. (2021) on a sample of 100 women patients diagnosed with breast cancer who underwent surgery, a higher average score was recorded in the BUT subscales ‘Concern for the Body Image’ and ‘Avoidance Conduct’. According to the authors, avoidance strategies, such as avoidable behavior in front of the mirror, avoidance of sexual activity or in relationships, were performed by women to deal with intrusive and distressing thoughts about their own body image. Women with breast cancer often undergo physical changes due to the disease itself or treatments such as surgery or radiotherapy, potentially affecting their self-esteem. An explanation for this hypothesis may lie in the objectification theory (Fredrickson et al., 2011). Indeed, women are often objectified and sexualized in the Western culture, and the body becomes the most visible part and main element of evaluation or judgment. As a result, breast cancer patients may fear negative feedback from the others due to changes in their body image, increasing shame, body avoidance and a negative attitude of self- objectification. Therefore, it becomes important to consider this risk for patients’ well-being in order to develop strategies promote self-acceptance.

In addition to these challenges for women, it should be noted that in the general population between a negative body image and a potentially increased risk of depressive thinking (Waite and Freeman, 2017; Núñez et al., 2021): excessive preoccupation with weight, shape and eating were found to be the main symptoms of body image avoidant behavior (Fairburn et al., 1999). Feelings of uncertainty and inadequacy are highly present in body image disorders (Bijsterbosch et al., 2022). The results of our study showed that body avoidance can be significantly associated with depressive symptoms. Previous research also highlighted a relationship between depression and body image (Di Cara et al., 2019). Significant associations were found between body weight dissatisfaction and depression in underweight, normal weight, overweight and obese individuals (Richard et al., 2016). Body-related avoidant behavior seems to lead to an increased sensitivity of the behavioral inhibition system, acting as a risk factor for the development of a patient’s psychological distress, and impacting different psychopathological dimensions (Struijs et al., 2017). Concerns regarding one’s appearance have been connected to higher levels of psychological distress and have been demonstrated to be a significant predictor of symptoms of anxiety and depression (Linardon et al., 2018). Cancer stigma, social support, social comparisons, and other coping strategies may lead to different health outcomes in patients as they can influence their adjustment process, increasing (as in the case of stigma) or decreasing (as in the case of supportive coping strategies) cancer-related distress (Kang et al., 2020; Gallagher-Squires et al., 2021). Indeed, women undergoing breast cancer treatment reported depressed feelings and reduced daily functioning as a result of these concerns (Iddrisu et al., 2020). According to our results, there is also a relationship between body avoidance and anxiety: those who report more avoidance in relation to their body seem to be more prone to develop anxiety symptoms (Bardeen et al., 2014). It was also discovered that the psychological aspect of hostility was connected to baseline body avoidance in the pre-operative period. The term ‘hostility’, on the other hand, refers to emotionally activated and aggressive behavior (Barata et al., 2016). For example, among teenagers, aggressiveness and body image dissatisfaction may be positively associated (Peng et al., 2022). In patients with cancer, hostility is often experienced as a manifestation of the psychological distress resulting from cancer diagnosis and progression (Caruso and Breitbart, 2020). Indeed, high levels of hostility in these patients are associated with maladaptive coping strategies, in particular the adoption of a pessimistic attitude toward the disease by increasing feelings of worry (Grassi et al., 2021).

Examining the differences in pre- and post-operative body image after surgery was the second goal of our research. Our findings revealed that the psychological symptoms addressed (hostility, anxiety, sadness, and somatization) were specifically associated with weight phobia. Weight phobia is a term coined by Crips to indicate the almost phobic avoidance of high-calorie foods and body weight observed in women with anorexia nervosa (Crips, 1967). According to the avoidance paradigm, ‘weight phobia’ would explain most of the behavioral expressions adopted to avoid aversive stimuli such as weight gain (Dalle Grave et al., 2019). This leads to excessive attention and preoccupation with one’s weight. Weight gain can have a negative impact on self-esteem, decrease the quality of life of women with breast cancer (Lankester et al., 2002) and contribute to increased body dissatisfaction (Friedman et al., 2002). Breast changes after surgery can lead to marked psychological distress in women since the breast is one aspect of a woman’s overall sense of femininity and body image (Helms et al., 2008) and women with breast cancer expressed a severe degree of distress after post-treatment weight gain (Halbert et al., 2008). Especially in relation to the dread of gaining weight Cash et al. (2004) noted that this issue was very prevalent in women having surgery. In particular, patients with premorbid concerns about body weight who had undergone mastectomies reported more feeling of depression and more suicidal ideation after surgery (Margolis et al., 1989). Gaining weight after breast cancer is typical and can significantly lower one’s quality of life (Vance et al., 2011). Along the same lines, researchers such as Figueiredo et al. (2004) found that women who placed high importance on physical appearance tended to endorse greater mental health difficulties and anxiety after cancer surgery. Body image concerns and weight phobia are significantly correlated with higher levels of anxiety and depression (Fobair et al., 2006), and worse quality of life (Falk Dahl et al., 2010). Concerns about body image and weight have been shown to have a greater impact on feelings of shame (Carter et al., 2022), which can lead to feelings of hostility toward oneself, anxiety, and depressive symptoms. Fobair et al. (2006) also found that cancer patients are most concerned about their body image and body weight in the immediate postoperative period and after completing other forms of treatment.

Overall, our findings demonstrated that weight phobia and body avoidance, two important components of body image, may have a significant influence on psychological symptoms. An increased risk of somatization, anxiety, depression, and hostility would result from higher levels of body avoidance dimensions (pre-surgery) and alterations in weight-phobia (post-surgery). The results of this study seem to be consistent with the literature, making them significant aspects to be considered to improve treatment adherence and the management of symptoms before, during and after surgery. In particular, our findings may shed light on clinical and research perspectives targeting body image concerns and symptom management in breast cancer patients through both conventional and novel psychological approaches. The most cutting-edge psychological intervention strategies involve virtual reality-based protocols, which are particularly effective in symptom management and rehabilitation for breast cancer patients (Zhang et al., 2022). These interventions can help patients cope with post-surgery changes in body image, with promising outcomes on overall well-being. Mindfulness or progressive muscle relaxation may mitigate postoperative distress (Parsa Yekta et al., 2017), and also these relaxation techniques may be delivered using virtual reality as an innovative tool to support patients in their cancer treatment path (Parsa Yekta et al., 2017; Pizzoli et al., 2019). A recent study conducted by Sebri et al. (2023) proposed a virtual reality intervention aimed at fostering patients’ emotional well-being. The protocol improved breast cancer survivors’ awareness of internal feelings, reducing negative emotions (including fear of recurrences), and body-related symptoms. Improvements in disease management with positive outcomes especially on anxiety-depressive symptoms may also depend on the type of virtual protocol used to support patients through tailored recommendations provided online (Zhang et al., 2022). By providing highly personalized support and psychoeducational strategies, healthcare providers may empower patients to navigate their emotional responses to body changes effectively. Combining these strategies ensures a holistic approach to body concerns in order to promote psychological health in breast cancer survivors.

### 4.1 Limitations

Despite its good clinical translatability, our study presents some limitations. The reliance on only two relatively proximate time points (i.e., pre-intervention and 2 months following surgery) may limit the comprehensive understanding of how individuals cope with the breast surgery process over an extended period. Indeed, the close timeframe adopted for psychological assessment might not capture the full spectrum of adjustments and changes patients undergo in a longer term post-surgery. Additionally, the relatively constrained sample size, specifically focusing on patients undergoing quadrantectomy and mastectomy due to breast carcinoma, might restrict the generalizability of the findings to a broader population. The nature of our cross-sectional design also limits the ability to establish a causal relationship between variables, only allowing for observations at distinct points rather than tracking changes over time within individuals. Another limitation is that data on whether patients were in metastatic or non-metastatic stages was not specifically collected. Consequently, it is unclear whether a mixed cohort influenced the results or if certain patterns emerged from specific diagnostic groups. This lack of detailed information limits the ability to generalize the findings and assess the impact of different stages of breast cancer on psychological outcomes. For further studies, it could be advised to consider further clinical and psychological variables and more time points to better elucidate the extent and variety of adjustments in breast cancer patients undergoing surgery.

## 5 Conclusion

A poorer body image has been correlated with physical and psychological distress (Paterson et al., 2016) and a decrease in quality of life, increasing the risk of recurrence and mortality for women with breast cancer (Montagnese et al., 2020). Many psychological consequences negatively impact women with breast cancer, and increased attention to the reduction and management of disease-related symptoms could improve their psychological well-being (Janz et al., 2007) as well as their treatment adherence (Dinapoli et al., 2021). This study sought to determine whether and how pre-post alterations in body image could affect the psychological effects of breast surgery, as well as the overall impact of body image on these outcomes. This work might thus pave the way for future approaches to designing psychological interventions focused on the unique needs of each patient and consider risk and protective variables in order to enhance psychological well-being. Even though the physical side effects of breast cancer treatment have been shown to cause long-term distress, it is crucial to foster future research to investigate adaptive coping mechanisms that may contribute to overcoming the psychological and social effects of breast cancer. One internal resource that may help in adapting to the physical changes due to cancer in terms of body image may be, for instance, self-compassion (Przezdziecki et al., 2013). Overall, body image issues may act as important predictors of quality of life (Ettridge et al., 2022), and improving body image-related distress should be a clinical priority to promote and prevent well-being in women with breast cancer (Pila et al., 2018).

## Data availability statement

The raw data supporting the conclusions of this article will be made available by the authors, without undue reservation.

## Ethics statement

The studies involving humans were approved by the Department of Education, Psychology, Communication (For.Psi.Com.), University of Bari Aldo Moro, Bari. Ethics reference code: ET-23-01. The studies were conducted in accordance with the local legislation and institutional requirements. The participants provided their written informed consent to participate in this study.

## Author contributions

VV: Methodology, Formal analysis, Data curation, Writing – review & editing, Writing – original draft. IP: Writing – review & editing, Software, Data curation, Formal analysis. CA: Writing – review & editing, Visualization, Formal analysis. MB: Writing – review & editing, Resources. CS: Writing – review & editing, Investigation, Resources. MM: Writing – review & editing, Validation, Data curation. MD: Writing – review & editing, Supervision. PT: Writing – review & editing, Supervision, Visualization. LA: Writing – review & editing, Supervision, Methodology. AT: Project administration, Methodology, Writing – review & editing, Writing – original draft, Supervision, Conceptualization.
